# The Key Role of RNA Modification in Breast Cancer

**DOI:** 10.3389/fcell.2022.885133

**Published:** 2022-06-01

**Authors:** Yang Liu, Tong Zhu, Yi Jiang, Jiawen Bu, Xudong Zhu, Xi Gu

**Affiliations:** Department of Oncology, Shengjing Hospital of China Medical University, Shenyang, China

**Keywords:** RNA modification, breast cancer, m^6^A, carcinogenesis, treatment

## Abstract

The modulation of the function and expression of epigenetic regulators of RNA modification has gradually become the hotspot of cancer research. Studies have shown that alteration of epigenetic modifications can promote the development and metastasis of breast cancer. This review highlights the progress in characterization of the link between RNA modification and the prognosis, carcinogenesis and treatment of breast cancer, which may provide a new theoretical basis for development of effective strategies for monitoring of breast cancer based on epigenetics.

## Introduction

Breast cancer is the most common female cancer in the world (Harbeck et al., 2019). Current treatments for breast cancer include surgery, chemotherapy, radiotherapy, hormone therapy and targeted therapy (Bray et al., 2018). However, comprehensive treatment strategies for breast cancer are still limited. Therefore, eradication of breast cancer remains a significant challenge, and there is an urgent need for new treatment strategies (Pedrosa et al., 2018). All biological macromolecules require post-synthesis and covalent modifications (Maresca and Wismayer, 2016). Over 100 different kinds of post-synthetic modifications have been identified to exist in RNA, and the four kinds of RNA bases and ribose can be targets for modification (He et al., 2019). Studies have shown that RNA can exert functional effects on the expression of gene in addition to acting as an effector of protein synthesis. Therefore, the importance of RNA modification has received increased attention, and studies have shown that dysregulation of RNA modification may be associated with human diseases, including breast cancer (Huang et al., 2020; Boccaletto et al., 2022). Herein, we review the progress of research focused on RNA modification and regulators of RNA modification in breast cancer.

## Ribonucleic Acid Modification Regulatory Proteins in Breast Cancer

Eight different internal RNA modifications associated with a variety of cancers have been characterized: methylation of adenosine on position 6 on RNA to generate N 6-methyladenosine (m^6^A); methylation of cytidine on position 5 to produce RNA with 5-methylcytosine (m^5^C); methylation at position 1 of adenosine on tRNA to formN-1-methyladenosine (m^1^A); 7-methylguanosine (m^7^G); pseudouridylation of RNA to produce pseudouridine; editing of RNA adenosine to inosine; U34 modification of tRNA; N4-acetylcytidine (ac^4^C) (Rong et al., 2021). Modification of RNA is a dynamic process that includes insertions, deletions, and recognition *via* specific cellular components called “writers,” “erasers” and “readers” respectively.

### N6-Methyladenosine Methyltransferases

Dynamic and reversible RNA modification plays a key role in maintaining RNA balance, and can affect splicing, translation, degradation, and localization of RNA, resulting in the regulation of various biological functions in human disease (Li and Mason, 2014). Ribosomal RNA (rRNA) and transport RNA (tRNA) are the two most abundant RNAs. Post-transcriptional modifications are very common on rRNA and tRNA (Frye et al., 2018). Continuous development of technology to detect RNA modifications has allowed for identification of post-transcriptional modifications of messenger RNA (mRNA) and non-coding RNA (ncRNA) (Zhao et al., 2017). N6-methyladenosine (m^6^A) is the most common mRNA modification in mammals. In addition, m^6^A was shown to play an important role in stem cell self-renewal, metabolism and metastasis in multiple cancers (Dong et al., 2021; Wood et al., 2021). The methyltransferase complex (MTC), also known as the m^6^A “writer,” catalyzes m^6^A modification of adenylate on mRNA, and includes methyltransferase-like 3 (METTL3), methyltransferase-like 14 (METTL14), Wilms tumor 1 associated protein (WTAP), RNA binding motif protein 15/15B (RBM15/15B), Cbl proto-oncogene like 1 (CBLL1), zinc finger CCCH type containing 13 (ZC3H13), KIAA1429, CCHC-type zinc finger protein (ZCCHC4) and methyltransferase-like 16 (METTL16). METTL3 is a major catalytic enzyme in the N6-adenine methyltransferase system. The expression level of METTL3 is not consistent in each subtype of breast cancer (Yang et al., 2020). It was reported to play a tumor-suppressive role in triple-negative breast cancer (TNBC) while play an oncogenic role in other subtypes (Shi et al., 2020; He et al., 2021; Ruan et al., 2021). METTL14 stabilizes METTL3 and recognizes target RNA, which is found to be an oncogene or a tumor suppressor gene in breast cancer (Gong et al., 2020; Sun et al., 2020). WTAP is the main regulatory component of the m^6^A methylation complex, and has mutual effects with METTL3 and METTL14 to aid in nuclear localization. The expression of WTAP varied in different in breast cancer studies (Wu et al., 2019; Wang et al., 2022). RBM15/RBM15B interacts with spliceosome components to participate in the modulation of m^6^A modification in a WTAP-dependent manner. RBM15 was identified to be significantly high in TNBC (Yang et al., 2020). ZC3H13 is critical for anchoring regulatory complex in the nucleus. It was recognized as a tumorsupressor which positively related with tumor infiltrating lymphocytes (TILs) in the breast cancer (Gong et al., 2020). KIAA1429 is essential in the methylation process as a candidate new subunit in the methylase complex. High expression of KIAA1429 was associated with a poor prognosis in breast cancer (Zhang et al., 2022). CBLL1, as a co-regulator of m^6^A methylation, was proved to promote the apoptosis in breast cancer (Zheng F. et al., 2021). ZCCHC4, a novel methyltransferase in the mediating of ribosome methylation, has a high expression in the breast lesion compared with pancancerous tissue (Pinto et al., 2020). METTL16 targets ncRNAs, lncRNAs and pre-mRNAs which is critical in splicing regulation (Su et al., 2022).

### N6-Methyladenosine Demethylases

The demethylases ALKB homolog 5 (ALKBH5) and fat mass and obesity-related protein (FTO), also known as “m^6^A erasers,” remove m^6^A using ferrous iron as a cofactor and α-ketoglutarate as a co-substrate (Jia et al., 2011; Zheng et al., 2013). ALKBH5 was higher in breast cancer tissue than in adjacent normal tissue of TNBC (Wang S. et al., 2020). FTO can oxidize m^6^A to N6-hydroxymethylsalicylic acid and N6-formyl adenosine, which can be hydrolyzed to adenine (Fu et al., 2013). The expression of FTO varied in different breast cancer studies. Most of studies show that down-regulation of FTO enhanced the phenotype of invasiveness, migration and EMT in breast cancer (Jeschke et al., 2021). But in other cases, FTO played an oncogenic role with a high expression in breast cancer (Niu et al., 2019).

### N6-Methyladenosine Readers

The “readers” mainly include the YTH domain family (YTHDF) and heterogeneous nuclear ribonucleoproteins (hnRNPs) family, Insulin-like growth factor 2 mRNA-binding proteins (IGF2BPs) and YTH domain-containing protein (YTHDC) increase the translation levels of modified RNAs which recognize m^6^A, bind RNA and participate in regulatory functions (Huang et al., 2018; Xing et al., 2019; Dai X.-Y. et al., 2021). The YTHDF family includes three paralogs YTHDF1, YTHDF2 and YTHDF3, which can also be referred to as DF1, DF2, and DF3. DF1 promotes mRNA translation, DF2 promotes mRNA degradation, and DF3 promotes translation and degradation (Zaccara and Jaffrey, 2020). YTHDF1 and YTHDF3 were also found to overexpress in breast cancer (Chen et al., 2022; Lin et al., 2022). IGF2BP 2/3 and YTHDC2 were highly expressed in basal-like breast cancer (Yang et al., 2020). The overexpression of hnRNPc were related to poor prognosis in patients (Lv et al., 2021a), but hnRNPc A2/B1 was reported to negatively regulate the metastasis of breast cancer (Liu Y. et al., 2020). Although various readers, writers, and erasers may be independently associated with numerous changes in signaling pathways of cancer, there is evidence that writers, erasers and readers may have interplay with each other in cancer. Regulators in the same functional category show significant genetic changes and highly correlated expression patterns in cancer (Li et al., 2019). In addition, m^6^A methylation was involved in regulation of the malignant phenotypes of tumors by controlling the expression of tumor-related genes in breast cancer (Barbieri and Kouzarides, 2020; Zhang et al., 2020). Recent studies have shown that m^5^C, m^1^A, m^7^G, and recently discovered ac^4^C modifications, also play important roles in RNA processing and metabolism. For example, m^5^C could promote enucleation of mRNA through binding to its reader protein Aly/REF export factor (ALYREF) (Yang et al., 2017), m^1^A can affect the translation efficiency of its modified mRNA (Li et al., 2017; Safra et al., 2017), and ac^4^C stabilizes its modified mRNA and enhances translation efficiency (Arango et al., 2018).

### 5-Methylcytosine

The m^5^C modification is involved in the metastasis and proliferation of cancer cells, and the development of cancer stem cells. The currently identified writers of m^5^C genes include NOP2/Sun RNA methyltransferase 2 (NSUN2), NSUN6, tRNA aspartic acid methyltransferase 1 (TRDMT1), tRNA-specific methyltransferase 4B (TRM4B) and OsNSUN2 (Bujnicki et al., 2004; Moon and Redman, 2014; Liu et al., 2017; Muller et al., 2019; Tang et al., 2020; Li H. et al., 2021). The “readers” include ALYREF, DNA repair protein RAD52 homolog (RAD52) and Y-box binding protein 1 (YBX1) (Yang et al., 2017; Chen et al., 2020; Xue et al., 2021).

### N1-Methyladenosine

The main modification of tRNA is m^1^A, which has also been found in 28SrRNA. The tRNA methyltransferase 10 homologue A (TRM)-TRM61 complex is the only known methyltransferase that catalyzes m^1^A modification (Saikia et al., 2010), and YTH protein family is a potential reader of m^1^A modifications (Dai et al., 2018). In addition, ALKBH3 is an eraser of m^1^A (Li et al., 2016).

### 7-Methylguanosine

The m^7^G modification was illustrated as part of the type O’ cap structure of mRNA and was also observed in rRNA and tRNA. The m^7^G maintained the integrity of structure mediated by the METTL1-WDR4 complex (Dai Z. et al., 2021). In addition, the m^7^G modification on rRNA is induced by Williams Beuren syndrome chromosome 22 region protein (WBSCR22) (Haag et al., 2015). Up-regulation of METTL1/WDR4 can promote the level of m^7^G modification on tRNAs, which in turn promotes the stability of tRNAs and the translation of mRNAs (Katsara and Schneider, 2021).

### Pseudouridine

Pseudouridine can maintain the structure and stability of tRNA. The most-studied regulatory factor related to pseudouridine modification is Dyskerin Pseudouridine Synthase 1 (DKC1), which is a component of a small nucleolar ribonucleoprotein complex, needs RNA guidance to exert its catalytic activity, is overexpressed in various types of cancer.

### Adenosine-to-Inosine Editing

Adenosine deaminases targeting RNA (ADARs) are effective in RNA editing, and are particularly important in the process of converting adenosine residues in double stranded RNA to creatinine (Ota et al., 2013). The ADAR1p110 subtype can regulate the stability of the chromosome terminal genome, and is required for continuous proliferation of cancer cells (Shiromoto et al., 2021).

### U34 on Transport Ribonucleic Acid

Establishment of the U34 modification results from three steps: modification of U34 with an extender complex to produce 5-carboxymethyluridine (cm5U), transformation of cm5u to 5-methoxycarbonylmethyluridine (mcm5U) mediated by ALKBH8. Finally, thiolase, cytoplasmic trna2 thiolated protein 1 (CTU1), and CTU2 promote the formation of 5-methoxycarbonylmethyl-2-thiouridine (mcm5s2U) on specific tRNA (tRNAUUULys, tRNArUCGlu and tRNAAUGln) (Rapino et al., 2017).

### N4-Acetylcytidine

N4-acetylcytodine (ac^4^C) is a conserved chemical modification in eukaryotes and prokaryotes. Early studies suggested that ac^4^C modifications mainly occurred on tRNA and 18SrRNA. Recent studies showed extensive ac^4^C modifications on mRNA, with similar abundance to the m^7^G cap modification on mRNA. To date, N-acetyltransferase 10 (NAT10) is the only protein known to have both an acetylase domain and an RNA-binding domain, and is therefore considered an RNA ac^4^C-modifying enzyme (Sas-Chen et al., 2020; Yang C. et al., 2021).

## Association of Ribonucleic Acid Modification and Breast Cancer Prognosis

Data from public databases and clinical studies have indicated that levels of RNA modification regulators have prognostic value for breast cancer (Zheng F. et al., 2021). Low expression of METTL3, METTL14, WTAP and FTO was shown to correlate with relapse-free survival in breast cancer (Wu et al., 2019). METTL3 was also demonstrated to be related with a poor survival rate in breast cancer (Wang H. et al., 2020). METTL14 and ZC3H13 were associated with favorable prognosis, and correlated with adenomatous polyposis coli (APC). Furthermore, ZC3H13, METTL14 and APC expression levels were positively related with the number of infiltrating immune cells in breast cancer (Gong et al., 2020). The regulators YTHDF1, YTHDF3 and KIAA1429 were found to be upregulated in breast cancer, and were associated with the metastasis of lymph nodes, breast cancer progression, and also were predictors of poor prognosis (Liu et al., 2019; Anita et al., 2020; He et al., 2021). The demethylase ALKBH5 was found to be associated with poor prognosis in patients with TNBC (Wang S. et al., 2020). FTO was associated with short survival in Her-2 positive breast cancer (Xu et al., 2020). Non-coding RNAs such as miRNA, lncRNA, and circRNA, can undergo m^6^A modification, which regulates their expression and function. Ten m^6^A-modified lncRNAs-LINC00571, ANKRD10- IT1, LINC00593, miR-205HG, CIRBP- AS1, BLACAT1, SUCLG2- AS1, SAMD12- AS1, BVES-AS1, a18SrRNA nd HOXB-AS1 were used to construct a prognostic score model, and may be potential predictors of survival in patients with TNBC (Wu et al., 2021). A prognostic risk model comprised of six m^6^A-regulated lncRNAs-Z68871.1, AL122010.1, AL138724.1,OTUD6B-AS1, AC090948.3 and eosinophil granule ontogeny transcript (EGOT) for high-risk patients with tumor-infiltrating immune cells, indicated that m^6^A-regulated lncRNAs may modulate the immune microenvironment in breast cancer (Lv et al., 2021b). High expression of the m^6^A regulator hnRNPC, and low expression of hsa-miR-944, are associated with advanced stage breast cancer and poor prognosis (Lv et al., 2021a). Basal-like subtypes and other breast cancer subtypes are associated with the m^6^A regulators YTHDC2, IGF2BP2, IGF2BP3 and RBM15, and luminal A and B subtypes are classified into two clusters according to the methylation status of these four regulators. In addition, cluster1 has been found to be associated with cell adhesion signaling pathways and immune-associated genes of TILs. Furthermore, cluster1 was related to poor prognosis among patients with stage II and luminal B of breast cancer. The accuracy of diagnosis and efficacy of treatment may be improved by using m^6^A regulators as biomarkers of different subtypes (Yang et al., 2020). These studies indicated that METTL3, METTL14, WTAP, FTO, ALKBH5, and other N6-methyladenosine-related lncRNAs were associated with progression of breast cancer, and may be prognostic indicators. Changes in expression and activity of m^6^A modulators may promote breast cancer progression (Chen and Du, 2019; Lv et al., 2021b; Zhang et al., 2021) (Table 1). Few studies have mentioned m^5^C modifications and breast cancer, and most have focused on NSUN2. It was reported that NSUN2 expression was associated with tumor stage and pathological subtype of breast cancer. The m^5^C RNA methylation regulators NSUN2 and NSUN6 were predictors of survival and affected the progression and tumor immune microenvironment in TNBC (Huang Z. et al., 2021). Low expression of DKC1, rRNA pseudouridine modification, and decreased intrinsic ribosomal activity are associated with better breast cancer prognosis (Elsharawy et al., 2020; Guerrieri et al., 2020). In addition, the U34 modification enzymes ELP3, CTU1, and CTU2 were shown to be upregulated in breast cancer (Delaunay et al., 2016) (Table 1). Determination of the predictive value of mRNA m^7^G and m^1^A modifications, editing of RNA adenosine to inosine, U34 modification of tRNA, or ac^4^C-related effectors for tumor prognosis require further study.

**TABLE 1:** The main role of regulators of RNA modification related with prognosis of breast cancer.

Gene	Type of regulator	Type of Modification	Role in survival	Role in tumor	Expression in cancer	References
METTL3	Writer	m^6^A	Poor favorable	Oncogene suppressor	Upregulated downregulated	Wang H et al. (2020), Wu et al. (2019)
METTL14	Writer	m^6^A	Favorable	Suppressor	Downregulated	Wu et al. (2019); Gong et al. (2020)
WTAP	Writer	m^6^A	Favorable	Suppressor	Downregulated	Wu et al. (2019)
RBM15	Writer	m^6^A	Poor	Oncogene	Upregulated	Yang et al. (2020)
ZC3H13	Writer	m^6^A	Favorable	Suppressor	Downregulated	Gong et al. (2020)
KIAA1429	Writer	m^6^A	Poor	Oncogene	Upregulated	Liu et al. (2019); Zhang et al. (2022)
CBLL1	Writer	m^6^A	Favorable	Suppressor	Downregulated	Zheng F et al. (2021)
ALKBH5	Eraser	m^6^A	Poor	Oncogene	Upregulated	Wang S et al. (2020)
FTO	Eraser	m^6^A	Poor Favorable	Oncogene suppressor	Upregulated Downregulated	Xu et al. (2020), Wu et al. (2019)
YTHDF1/3	Reader	m^6^A	Poor	Oncogene	Upregulated	Anita et al. (2020); He et al. (2021)
hnRNPC	Reader	m^6^A	Poor	Oncogene	Upregulated	Lv et al. (2021a)
hnRNPC A2B1	Reader	m^6^A	Favorable	Suppressor	Downregulated	Liu Y et al. (2020)
IGF2BP2	Reader	m^6^A	Poor	Oncogene	Upregulated	Yang et al. (2020)
IGF2BP3	Reader	m^6^A	Poor	Oncogene	Upregulated	Yang et al. (2020)
YTHDC2	Reader	m^6^A	Poor	Oncogene	Upregulated	Yang et al. (2020)
NSUN2	Writer	m^5^C	Poor	Oncogene	Upregulated	Huang Z et al. (2021)
NSUN6	Writer	m^5^C	Favorable	Suppressor	Downregulated	Huang Z et al. (2021)
DKC1	Writer	Pseudouri dine	Favorable	Suppressor	Downregulated	Elsharawy et al. (2020)
ELP3	Writer	U34	Poor	Oncogene	Upregulated	Delaunay et al. (2016)
CTU1	Writer	U34	Poor	Oncogene	Upregulated	Delaunay et al. (2016)
CTU2	Writer	U34	Poor	Oncogene	Upregulated	Delaunay et al. (2016)

## Roles of the Ribonucleic Acid Modification in the Carcinogenesis of Breast Cancer

Previous studies have proven that m^6^A levels were strongly associated with cancer, which indicated that m^6^A may play a crucial role in the occurrence or inhibition of malignant tumors (Helm and Motorin, 2017; Mohammad et al., 2019; Gu et al., 2020).

### Ribonucleic Acid Modification Regulators in the Proliferation, Invasion and Metastasis of Breast Cancer

**FIGURE 1:**
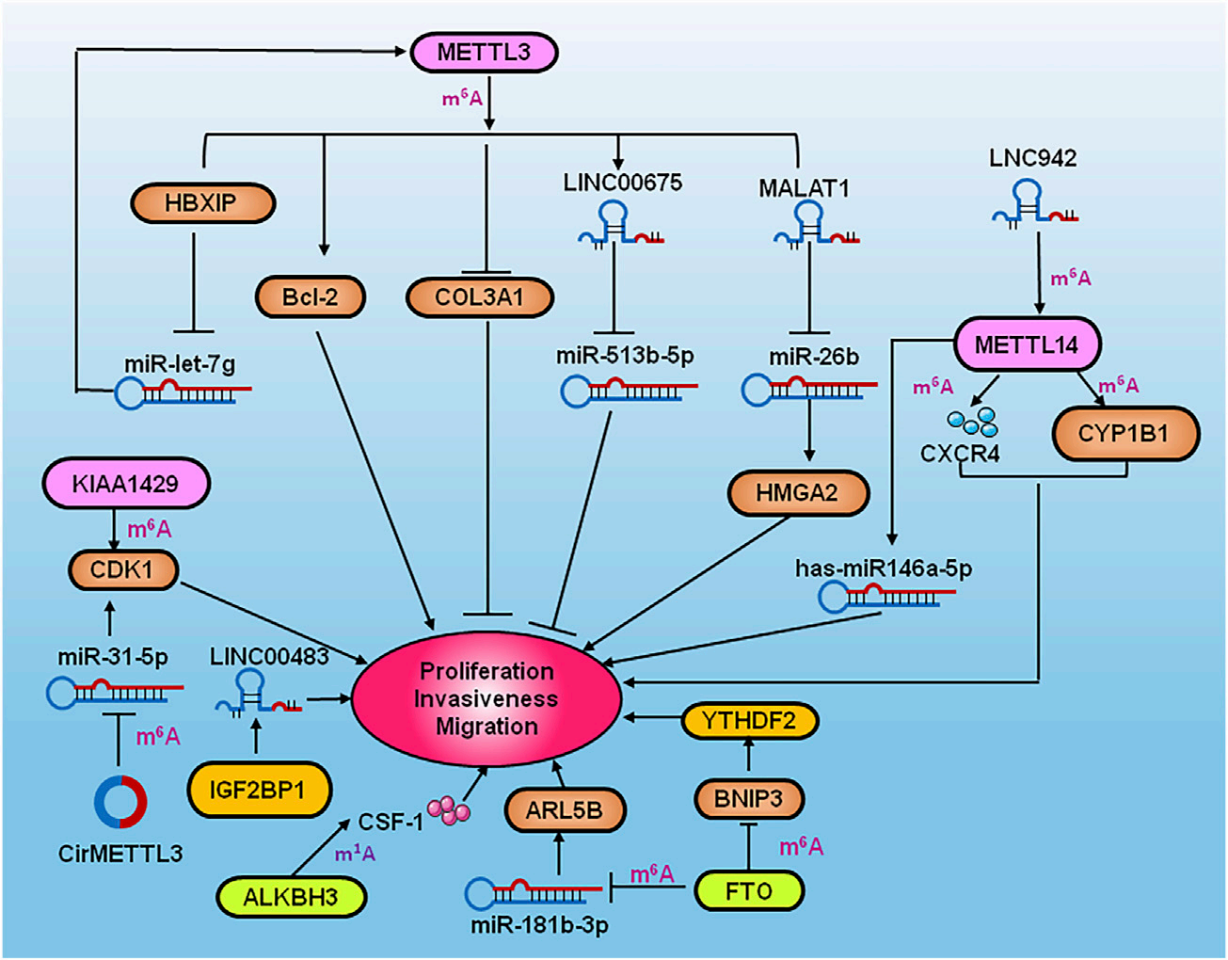
Diagram of RNA modification regulators playing a vital role in the proliferation, invasion and metastasis of breast cancer. METTL3 promoted the proliferation, invasion and metastasis by targeting Bcl-2, while it reduced the expression of COL3A1 to inhibit the metastasis of breast cancer. LINC00675 m^6^A methylation induced by METTL3 resulted in the inhibiting miR-513b-5p to suppress malignant phenotype breast cancer. LncMALAT1 increased/HMGA2 to facilitate the proliferation, invasion and metastasis of breast cancer cells by inhibiting miR-26b. LNC942 directly bound to METTL14 resulting in regulation of m^6^A methylation of CXCR4 and CYP1B1 and mediate the onset and development of breast cancer. METTL14 also increased the expression of has-miR-146a-5p to promote invasion and migration of breast cancer. The writer KIAA1429 promoted the proliferation and metastasis of breast cancer by regulating CDK1, whereas circMETTL3 promoted the progression of breast cancer cells by targeting miR-31-5p/CDK1. FTO enhanced ARL5B by down-regulating miR-181b-3p to promote the invasion and migration of breast cancer. It also mediated m^6^A demethylation by YTHDF2 to enhance the proliferation and metastasis of breast cancer *via* inhibiting BNIP3. IGF2BP1 promote proliferation of breast cancer by binding to LINC00483. ALKBH3 induced the decay of CSF-1 to promote breast cancer cell invasiveness.

The writer KIAA1429 promotes proliferation and metastasis of breast cancer by modulating cyclin-dependent kinase 1 (CDK1) (Qian et al., 2019). Studies showed that the increasing of METTL3 promoted proliferation and inhibited apoptosis in breast cancer by targeting Bcl-2 (Wang H. et al., 2020). Hepatitis B X-interacting protein (HBXIP) upregulated the expression of METTL3 by inhibiting the miRNA let-7g in another study. In addition, METTL3 activated HBXIP *via* m^6^A modification, which promoted cell proliferation in breast cancer as part of a positive feedback loop (Cai et al., 2018). On the contrary, METTL3 played an anti-tumor role by COL3A1 and circMETTL3/miR-34c-3p in TNBC (Shi et al., 2020; Ruan et al., 2021). The expression of circMETTL3 was also found to be increased in breast cancer, and promoted migration, proliferation and invasion of breast cancer cells by targeting miR-31-5p/CDK1 (Li Z. et al., 2021). A further study showed that the m^6^A levels were significantly upregulated in lung metastatic breast cancer cells, which promoted the translation, elongation, and mRNA stability of keratin 7 (KRT7), a key epithelial-to-mesenchymal transition (EMT)-associated protein, by targeting FTO and METTL3, thereby promoting lung metastasis of breast cancer cells. LINC00675 m^6^A methylation was increased by METTL3, which resulted in the interaction with miR-513b-5p and inhibition of cancerous properties of breast cancer (Fan and Wang, 2021). LNC942 directly bound to METTL14 and promoted the expression of METTL14 protein through a specific binding domain (+176 to +265), resulting in the regulation of m^6^A methylation of C-X-C motif chemokine receptor 4 (CXCR4) and cytochrome P450 family 1 subfamily B member 1 (CYP1B) to stabilize their expression and translation and mediate the onset and development of breast cancer (Sun et al., 2020). It was showed that METTL14 increased the expression of has-miR-146a-5p and promoted the invasion and migration of breast cancer (Yi et al., 2020). High level of FTO enhanced the expression of ARL5B by down-regulating miR-181b-3p to promote the invasion and migration of Her-2 positive breast cancer (Xu et al., 2020). FTO mediated m^6^A demethylation in a YTHDF2-dependent manner and promoted the proliferation and metastasis of breast cancer *via* inhibiting BCL2 interacting protein 3 (BNIP3) (Niu et al., 2019). IGF2BP1 was shown to bind to LINC00483 and promote the proliferation of breast cancer cells (Qiao et al., 2021). Furthermore, the overexpression of NSUN2 induced by DNA hypomethylation promoted the proliferation, invasiveness and migration of breast cancer cells (Yi et al., 2017). Little is known about the functional mechanisms of m^1^A-modified RNA. Therefore, epigenetic transcriptome research should focus on the function of m^1^A-modified RNA. The up-regulation of m^1^A demethylase ALKBH3 was shown to be involved in decay of macrophage-colony stimulating factor-1 (CSF-1) mRNA, which resulted in promoting breast cancer cell invasiveness (Woo and Chambers, 2019) (Figure 1).

### Ribonucleic Acid Modification Regulators in the Breast Cancer Stem-Like Cells, Metastasis, Epithelial-to-Mesenchymal Transition, Glycosis and Immune Escape of Breast Caner

**FIGURE 2:**
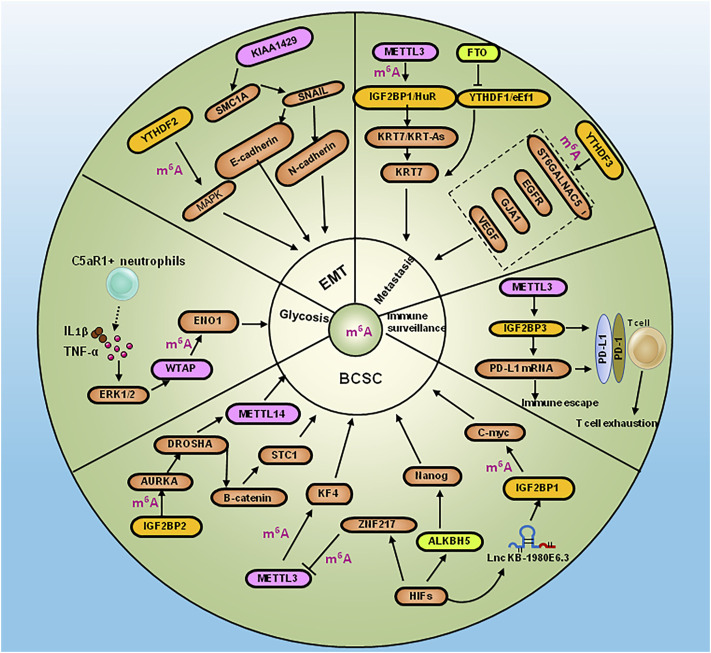
Diagram summarizing RNA modification regulators in the pathogensis of BCSC, metastasis, EMT, glycosis and immune escape of breast caner. BCSC: Binding of AURKA to DROSHA transcripts induced by IGF2BP2 to enhance BCSC stemness, meanwhile DROSHA interacted with β-catenin to contribute to the BCSC property by activating STC1. ALKBH5 or ZNF21 increased Nanog and KLF4 mRNA expression by m^6^A methylation, which led to pluripotency of breast cancer under hypoxic condition. The Lnc RNA KB-1980E6.3 facilitated BCSC self-renewal by IGF2BP1/c-Myc axis under hypoxic condition. Metastasis:YTHDF3 enhanced translation of ST6GALNAC5, GJA1, EGFR and VEFG to promote breast cancer metastasis to the brain. METTL3 and FTO promoted lung metastasis of breast cancer by KRT7 *via* suppressing YTHDF1/eEf1 complex and increasing IGF2BP1/HuR complex. EMT: YTHDF2 induced EMT by activating MAPK pathway. KIAA1429 promoted EMT by SMC1A/SNAIL in breast cancer. Glysosis: C5aR1-positive neutrophils secreted IL-1β and TNFα to synergistically activate ERK1/2, which resulted in the stabilizing WTAP to affect the glycolysis of breast cancer *via* ENO1. Immune surveillance: METTL3 upregulate PD-L1 *via* IGF2BP3 by m^6^A-dependent manner to modulate immune escape and T cell exhausition in breast cancer.

METTL3 was shown to methylate adenine 877 on the antisense nucleotide chain KRT7-AS of KRT7, which was recognized by IGF2BP1 and recruited the effector molecule HuR to increase the stability of the KRT7 and KRT7-AS complexes (Chen et al., 2021). METTL3 was demonstrated to upregulate PD-L1 expression *via* IGF2BP3 by m^6^A-dependent manner to modulate immune surveillance in breast cancer (Wan et al., 2022). The high level of METTL3 induced EMT, invasion and migration by targeting MALAT1/miR-26b/HMGA2 axis (Li et al., 2022). DROSHA RNase III was upregulated in a number of cancers and interacted with β-catenin to activate stanniocalcin 1 (STC1) in an RNA cleavage-independent manner, which in turn contributed to the properties of breast cancer stem-like cells (BCSCs). Aurora kinase A (AURKA)-induced m^6^A modification in BCSCs enhanced DROSHA mRNA stability. In addition, AURKA stabilized METTL14 by inhibiting its ubiquitination and degradation, thereby promoting methylation of DROSHA mRNA. Furthermore, binding of AURKA to DROSHA transcripts induced by IGF2BP2 to stabilize m^6^A-modified DROSHA, which enhanced BCSC stemness (Peng et al., 2021). Complement C5a receptor 1 (C5aR1)-positive neutrophils secreted IL (Interleukin) 1β and tumor necrosis factor α (TNFα) to synergistically activate ERK1/2, which resulted in phosphorylation of WTAP at serine 341, thereby stabilizing WTAP protein to promote RNA m^6^A methylation of enolase 1 (ENO1) and affected the glycolysis of breast cancer cells (Ou et al., 2021). The overexpression of writer KIAA1429 was shown to bind the 3′-UTR of structural maintenance of chromosomes 1A (SMC1A) to promote EMT in breast cancer (Zhang et al., 2022). Down-regulation of FTO was shown to increase adenine methylation at position 950 on KRT7 mRNA, and enhanced the elongation efficiency of translation by recruiting the effector molecule eEF-1 through the recognition protein YTHDF1. The overexpression of FTO and knockdown of METTL3 and KRT7 reduced lung metastasis (Chen et al., 2021). ALKBH5 or ZNF217 mediated demethylation of m^6^A in Nanog and KLF4 mRNA. The depleting of ALKBH5 reversed the pluripotency of breast cancer by inhibiting Nanog under hypoxic condition (Zhang et al., 2016). YTHDF3 enhanced the translation of m^6^A-enriched transcripts of ST6 beta-galactoside alpha-2, 6-sialyltransferase 5 (ST6GALNAC5), gap junction protein alpha 1 (GJA1), epidermal growth factor receptor (EGFR) and vascular endothelial growth factor (VEGF), which promoted breast cancer metastasis to the brain (Chang et al., 2020). Apoptosis was shown to be triggered by the inhibition of YTHDF2-dependent mRNA degradation in TNBC through MAPK pathway-dependent induction of the EMT, and increased the global translation of mRNA synthesis in MYC-driven breast cancers (Einstein et al., 2021). The Lnc RNA KB-1980E6.3 facilitated BCSC self-renewal and carcinogenesis under hypoxic condition. In addition, IGF2BP1 was shown to be recruited by LncRNA KB-1980E6.3 to strengthen the stability of c-Myc mRNA (Zhu et al., 2021). A study showed that CircBACH2 sponged hsa-miR-944, which resulted in MAPK signaling pathway-dependent up-regulation of hnRNPC expression and promotion of breast cancer cell proliferation (Lv et al., 2021a) (Figure 2).

The effects of RNA modification of target genes on progression of breast cancer depends on three factors: 1) the gene is a suppressor or an oncogene; 2) abnormal levels of RNA methylation in cancer; 3) Regulation of target mRNA modification. Taken together, the current study of m^6^A RNA methylation in tumors is still at an early stage. RNA modification and its regulators seem to act as a “double-edged sword” in the tumor development, so it is challenging to rationally interpret the controversial findings. It is the functional versatility and tunability of this modification that underscores the important role of the environment in biological process. Therefore, the function of RNA modification may be more complex and extensive than the existing reports, and further exploration of its role in different cancers is expected to provide in-depth insights into tumorigenesis and development.

## Ribonucleic Acid Modifications as Potential Drug Targets in Breast Cancer

Modification of RNA connects epigenetic transcriptomics with tumorigenesis and progression, and affects the processes of stem cell self-renewal and differentiation, proliferation and apoptosis, invasion and metastasis, drug resistance, and immunosuppression. Therefore, the key proteins involved in RNA modification are expected to become potential molecular targets for cancer diagnosis and treatment. To date, a number of small-molecule inhibitors that specifically target regulators of RNA methylation have shown great potential for suppression of carcinogenesis. For example, METTL3, METTL14 and WTAP were shown to be predictors of response to chemotherapy and hormone treatment (Song et al., 2021). S-adenosylhomocysteine (SAH) can be hydrolyzed to produce adenosine (adenine) and homocysteine, which can inhibit cellular methyltransferase activity through substrate inhibition, and regulates transmethylation through inhibition of METTL3-METTL14 activity (Eckert et al., 2019). The expression of MALAT1 was shown to be enhanced by METTL3 through recruitment of E2F transcription factor 1 (E2F1), resulting in transcription of anterior gradient 2 (AGR2), and subsequent adriamycin resistance in breast cancer (Li et al., 2022). In a further study, METTL3 also promoted maturation of miRNA-221-3p in an m^6^A-dependent manner, which negatively regulated HIPK2, upregulated the target gene Che-1, and induced chemoresistance of breast cancer cells to doxorubicin (Pan et al., 2021).

Adenylate kinase 4 (AK4) and the m^6^A writer METTL3 are highly expressed in tamoxifen-resistant breast cancer cell lines, and METTL3 was shown to promote tamoxifen resistance in breast cancer by promoting AK4 expression, reducing the production level of reactive oxygen species (ROS), and decreasing the activity of p38 (Liu X. et al., 2020). Metformin was found to inhibit the proliferation of breast cancer cells through upregulation of P21 in an m^6^A-dependent manner *via* METTL3 (Cheng et al., 2021). STM2457 is an orally bioavailable small molecule METTL3 inhibitor that are slated for human clinical trials by targeting a novel mechanism for the treatment of acute myeloid leukemia and other solid and hematological cancers (Yankova et al., 2021). In addition, WTAP binds to the m^6^A modified site of lncRNA DLGAP1 antisense RNA 1 (DLGAP1-AS1) to sponge miR-299-3p, resulting in adriamycin resistance in breast cancer (Huang T. et al., 2021). The inhibitor of 2-oxoglutarate oxygenase (OG) oxidase, IOX1, significantly inhibited ALKBH5 activity. Protein arginine methyltransferase 5 (PRMT5) inhibits doxorubicin-treated RNA m^6^A modification by promoting nuclear translocation of ALKBH5 (Wu et al., 2022). The applying of PRMT5 inhibitor tadalafil improves the chemosensitivity of Doxorubicin in breast cancer by modulating RNA methylation (Wu et al., 2022). The most widely studied RNA methylation regulator is FTO (Chen and Du, 2019). A few potent inhibitors of FTO have been reported in the literature, namely FG-2216/IOX3, FB23-2, rhein, meclofenamic acid (MA), entacapone, bisantren and brequinar (Mcmurray et al., 2015; Van Der Werf and Jamieson, 2019; Su et al., 2020; Xiao et al., 2020; Yang B. et al., 2021; Lv et al., 2022). FTO was shown to promote tumor glycolysis and limit the response of T cells. The FTO inhibitor Dac51 increased CD8+ T cell infiltration and acted in synergy with anti-PD-L1 blockade (Liu Y. et al., 2021). MA is a highly selective FTO inhibitor relative to ALKBH5 by using high-throughput fluorescence polarization analysis (Zheng Q.-K. et al., 2021). The overexpression of m^6^A reader hnRNPA2B1 (A2B1) resulted in tamoxifen and fulvestrant resistance, and decreased migration and invasion in TAM-resistant cells through activation of the protein kinase B (AKT) and mitogen-activated protein kinase (MAPK) signaling pathways (Petri et al., 2021). Transcription factor 3 (ATF3) was highly expressed in tamoxifen-resistant breast cancer, and was regulated by low expression of YTHDF2. Moreover, ATF3 enhanced the expression of ATP binding cassette subfamily B member 1 (ABCB1), which promotes tamoxifen resistance (Liu X. et al., 2021).

Through regulation by NSUN2, m^5^C modifications were shown to be involved in the onset of various cancers, and may be potential targets for cancer treatment (Huang Z. et al., 2021; Hu et al., 2021). The expression of NSUN2 could be reduced by the inhibition of sphingosine kinase (SPHK), which is involved in sphingolipid metabolism in cell growth. Therefore, the SPHK1 inhibitor SK1 may be a latent drug for treatment through modulation of NSUN2 expression (Guo et al., 2021). In addition, the m^5^C “reader” Y-box-binding-protein 1 (YBX1) is highly expressed in certain cisplatin-resistant cancers. A study showed that the YBX1 phosphorylation inhibitors including TAS0612 (multikinase inhibitor) and everolimus (rapamycin complex 1 inhibitor) mitigated antiestrogen resistance in breast cancer (Shibata et al., 2020). However, the effects of YBX1 inhibitors on drug resistance in breast cancer require further investigation (Jiang et al., 2022). Three inhibitors were developed based on the interaction between DKC1 and TERC to inhibit telomerase activity in breast cancer cell lines, which may aid in development of pseudouridine synthase inhibitors for treatment of cancer (Armando et al., 2018).

**FIGURE 3:**
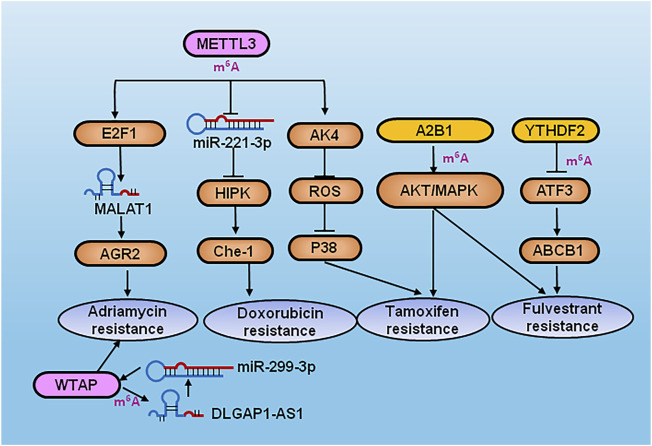
Diagram of RNA modification regulators inducing drugs resistance in breast cancer. METTL3 increased the expression of MALAT1 to activate AGR2 by recruiting E2F1 and subsequent adriamycin resistance in breast cancer. METTL3 also promoted maturation of miRNA-221-3p in a m^6^A-dependent manner and negatively regulated HIPK2 and upregulated Che-1 which induced chemoresistance of breast cancer cells to doxorubicin. METTL3 promoted tamoxifen resistance by promoting AK4 expression, reducing the production of ROS and decreasing the activity of p38. WTAP binds to DLGAP1-AS1 and sponged miR-299-3p to confer adriamycin resistance in breast cancer. A2B1 induced tamoxifen and fulvestrant resistance by AKT/MAPK signaling pathways. YTHDF2 downregulated ATF3 to activate ABCB1, resulting in tamoxifen resistance of breast cancer.

Modulating abnormal RNA modification levels can inhibit the occurrence and development of tumors (Figure 3). Although some RNA modification enzyme inhibitors have shown potential inhibitory effects in a variety of cancers (Table 2), more drugs and new therapeutic strategies related to RNA modification remain to be explored and requested in the clinical trials.

**TABLE 2:** The potential drugs applied in the treatment of breast cancer based on the RNA modification.

Drug	Target regulators	Modification	Target	References
S-adenosylhomocysteine (SAH)	METTL3-METTL14	m^6^A	Inhibit the activity of methyltransferase	Eckert et al. (2019)
Metformin	METTL3	m^6^A	Inhibit the proliferation of breast cancer cell by upregulating P21	Cheng et al. (2021)
STM2457	METTL3	m^6^A	Inhibit METTL3	Yankova et al. (2021)
Tadalafil	ALKBH5	m^6^A	Inhibit doxorubicin-induced RNA methylation	Wu et al. (2022)
FB23-2	FTO	m^6^A	Inhibit FTO	Van Der Werf and Jamieson, (2019)
FG-2216/IOX3	FTO	m^6^A	Inhibit FTO	Yang B et al. (2021)
Rhein	FTO	m^6^A	Inhibit FTO	Lv et al. (2022)
Entacapone	FTO	m^6^A	Inhibit FTO	Mcmurray et al. (2015)
MA	FTO	m^6^A	Inhibit FTO	Xiao et al. (2020)
Bisantren	FTO	m^6^A	Inhibit FTO	Su et al. (2020)
Brequinar	FTO	m^6^A	Inhibit FTO	Su et al. (2020)
Dac51	FTO	m^6^A	Increased CD8+ T cell infiltration and synergistic effect with anti-PD-L1 blockade	Liu Y et al. (2021)
SPHK	NSUN2	m^5^C	Maintained the metabolic balance of sphingolipids	Guo et al. (2021)
TAS0612	YBX1	m^5^C	Overcome anti-estrogen resistance	Shibata et al. (2020)
Everolimus	YBX1	m^5^C	Overcome anti-estrogen resistance	Shibata et al. (2020)

## Future Directions

Research on tumor-related RNA modification is still in its infancy. Increasing number of novel RNA modifications are gradually discovered, such as RNA glycosylation modification, which is remarkably suggested that glycoRNA may play an important role in physiological and pathological processes including host immune defense, tumor immune escape, and autoimmune diseases (Flynn et al., 2021). It is also necessary to develop new technologies to discover new type of RNA modification. Further studies on the role of RNA methylation in the immune response will provide broader prospects for immunotherapy and prevention of tumor drug resistance. In terms of clinical application, it is of great significance to continue to explore whether RNA modification-related proteins could be potential diagnostic and therapeutic targets. Development of more specific and effective regulators of RNA modification is expected to result in new options for tumor treatment. In the context of disease treatment, small molecule inhibitors that can target RNA methylation-related effector proteins may have great promise. Demonstration of preclinical efficacy of these targeted drugs may result in future clinical use of RNA epigenetic drugs.

## Conclusion

RNA methylation has been shown to exert tumor-promoting or tumor-suppressive activities, and is involved in the onset, development, and metastasis of breast cancer. The critical role of tumor-specific effects of RNA methylation provides insights into prognosis, pathogenesis, and treatment response in breast cancer. Design of novel therapeutics through targeted RNA modifications is an international research hotspot and may have profound implications in translational medicine application in breast cancer.

## Glossary

APC: adenomatous polyposis coli
ABCB1: ATP binding cassette subfamily B member 1
ARL5B: ADP ribosylation factor like GTPase5B
AGR2: anterior gradient 2
AK4: adenylate kinase 4
ADARs: adenosine deaminases acting on RNA
ALKBH5: Demethylases ALKB homolog 5
**ac** ^**4**^ **C**: N4-acetylcytodine
AURKA: aurora kinase a
ATF3: transcription factor 3
BNIP3: BCL2 Interacting Protein 3
C5aR1: C5a Receptor 1
COL3A1: collagen type III alpha 1 chain
CTU1: cytoplasmic trna2 thiolated protein 1
CDS: coding sequence
CDK1: cyclin-dependent kinase 1
CYP1B1: cytochrome p450 family 1 subfamily B member 1
CSF-1: macrophage-colony stimulating factor-1
DLGAP1-AS1: lncRNA DLGAP1 antisense RNA 1
eIF3: eukaryotic initiation factor 3
EGFR: epidermal growth factor receptor
EMT: epithelial-to-mesenchymal transition
ENO1: enolase 1
EGOT: eosinophil granule ontogeny transcript
E2F1: E2F transcription factor 1
FTO: Fat mass and obesity-related protein
HBXIP: hepatitis B X-interacting protein
hnRNP: heterogeneous nuclear ribonucleoprotein
IL: interlukin
IGF2BP1: insulin-like growth factor 2 mRNA-binding protein 1
KRT7: keratin 7
NSUN2: NOP2/sun RNA methyltransferase 2
GJA1: gap junction protein alpha 1
GRM3: glutamate metabotropic receptor 3
MALAT1: metastasis associated lung adenocarcinoma transcript 1
MTC: methyltransferase complex
METTL3: methyltransferase-like 3
METT14: methyltransferase-like 14
MAPK: mitogen-activated protein kinase
OG: oxoglutarate oxygenase
rRNA: ribosomal RNA
tRNA: transport RNA
mRNA: messenger RNA
ncRNA: non coding RNA
**m** ^**6**^ **A**: N6-methyladenosine
3’UTR: 3’untranslated region
mcm5s2U: 5-methoxycarbonylmethyl-2-thiouridine
RBM15: RNA binding motif protein 15
STC1: stanniocalcin 1
TNBC: triple-negative breast cancer
TNF: tumor-necrosis-factor
ROS: reactive oxygen species
SMC1A: chromosomes 1a
ST6GALNAC5: ST6 beta-galactoside alpha-2,6-sialyltransferase
RAD52: DNA repair protein RAD52 homolog
SAH: S-adenosylhomocysteine
SPHK: sphingosine kinase
TRM: tRNA methyltransferase 10 homologue A
WATP: wilms tumor 1 associated protein
ZC3H13: zinc finger CCCHType Containing 13
METTL16: methyltransferase like 16
YTHDF: YTH domain family
YTHDC: YTH domain-containing protein
TRDMT1: tRNA aspartic acid methyltransferase 1
TRM4B: tRNA-specific methyltransferase 4B
YBX1: Y-box binding protein 1
WBSCR22: Williams Beuren syndrome chromosome 22 region protein
YBX1: Y-box binding protein 1
